# Tissue stiffness at the human maternal–fetal interface

**DOI:** 10.1093/humrep/dez139

**Published:** 2019-10-03

**Authors:** Yassen Abbas, Alejandro Carnicer-Lombarte, Lucy Gardner, Jake Thomas, Jan J Brosens, Ashley Moffett, Andrew M Sharkey, Kristian Franze, Graham J Burton, Michelle L Oyen

**Affiliations:** 1 The Nanoscience Centre, Department of Engineering, University of Cambridge, Cambridge CB3 0FF, UK; 2 Department of Pathology, University of Cambridge, Cambridge CB2 1QP, UK; 3 Centre for Trophoblast Research, University of Cambridge, Cambridge CB2 3EG, UK; 4 Department of Physiology, Development and Neuroscience, University of Cambridge, Cambridge CB2 3EG, UK; 5 John Van Geest Centre for Brain Repair, Department of Clinical Neurosciences, University of Cambridge, Cambridge CB2 0PY, UK; 6 Warwick Medical School, University of Warwick, Coventry CV4 7AL, UK; 7 Department of Engineering, East Carolina University, Greenville, NC 27858-4353, USA

**Keywords:** mechanics, trophoblast invasion, blastocyst implantation, tissue stiffness, human

## Abstract

**STUDY QUESTION:**

What is the stiffness (elastic modulus) of human nonpregnant secretory phase endometrium, first trimester decidua, and placenta?

**SUMMARY ANSWER:**

The stiffness of decidua basalis, the site of placental invasion, was an order of magnitude higher at 10^3^ Pa compared to 10^2^ Pa for decidua parietalis, nonpregnant endometrium and placenta.

**WHAT IS KNOWN ALREADY:**

Mechanical forces have profound effects on cell behavior, regulating both cell differentiation and migration. Despite their importance, very little is known about their effects on blastocyst implantation and trophoblast migration during placental development because of the lack of mechanical characterization at the human maternal–fetal interface.

**STUDY DESIGN, SIZE, DURATION:**

An observational study was conducted to measure the stiffness of *ex vivo* samples of human nonpregnant secretory endometrium (*N* = 5) and first trimester decidua basalis (*N* = 6), decidua parietalis (*N* = 5), and placenta (*N* = 5). The stiffness of the artificial extracellular matrix (ECM), Matrigel®, commonly used to study migration of extravillous trophoblast (EVT) in three dimensions and to culture endometrial and placental organoids, was also determined (*N* = 5).

**PARTICIPANTS/MATERIALS, SETTING, METHODS:**

Atomic force microscopy was used to perform *ex vivo* direct measurements to determine the stiffness of fresh tissue samples. Decidua was stained by immunohistochemistry (IHC) for HLA-G+ EVT to confirm whether samples were decidua basalis or decidua parietalis. Endometrium was stained with hematoxylin and eosin to confirm the presence of luminal epithelium. Single-cell RNA sequencing data were analyzed to determine expression of ECM transcripts by decidual and placental cells. Fibrillin 1, a protein identified by these data, was stained by IHC in decidua basalis.

**MAIN RESULTS AND THE ROLE OF CHANCE:**

We observed that decidua basalis was significantly stiffer than decidua parietalis, at 1250 and 171 Pa, respectively (*P* < 0.05). The stiffness of decidua parietalis was similar to nonpregnant endometrium and placental tissue (250 and 232 Pa, respectively). These findings suggest that it is the presence of invading EVT that is driving the increase in stiffness in decidua basalis. The stiffness of Matrigel® was found to be 331 Pa, significantly lower than decidua basalis (*P* < 0.05).

**LARGE SCALE DATA:**

N/A

**LIMITATIONS, REASONS FOR CAUTION:**

Tissue stiffness was derived by *ex vivo* measurements on blocks of fresh tissue in the absence of blood flow. The nonpregnant endometrium samples were obtained from women undergoing treatment for infertility. These may not reflect the stiffness of endometrium from normal fertile women.

**WIDER IMPLICATIONS OF THE FINDINGS:**

These results provide direct measurements of tissue stiffness during the window of implantation and first trimester of human pregnancy. They serve as a basis of future studies exploring the impact of mechanics on embryo implantation and development of the placenta. The findings provide important baseline data to inform matrix stiffness requirements when developing *in vitro* models of trophoblast stem cell development and migration that more closely resemble the decidua *in vivo*.

**STUDY FUNDING/COMPETING INTEREST(S):**

This work was supported by the Centre for Trophoblast Research, the Wellcome Trust (090108/Z/09/Z, 085992/Z/08/Z), the Medical Research Council (MR/P001092/1), the European Research Council (772426), an Engineering and Physical Sciences Research Council Doctoral Training Award (1354760), a UK Medical Research Council and Sackler Foundation Doctoral Training Grant (RG70550) and a Wellcome Trust Doctoral Studentship (215226/Z/19/Z).

## Introduction

Our understanding of how mechanical cues direct molecular signaling pathways in the developing embryo is evolving. However, less is known on the role mechanics plays when the embryo implants into the endometrium. This tissue is transformed into decidua under the influence of progesterone, and two principal subtypes are recognized based on their position relative to the implantation site. The decidua basalis lies directly under the developing placenta and is invaded by the trophoblast cells, whereas the decidua parietalis lies on the opposite side of the uterus and is not involved in the process of placentation. Interactions between trophoblast cells and the decidua basalis are critical for normal placental development. Human trophoblast differentiates along two main pathways, villous and extravillous (EVT) (Apps *et al.*, 2011). EVT cells originate at the tip of the placental anchoring villi and invade into the decidua, the uterine lining during pregnancy. Their physiological role is to remodel the maternal spiral arteries to ensure an adequate maternal blood supply for normal fetal growth and development (Burton *et al.*, 2009). Shallow invasion is associated with the ‘great obstetrical syndromes’ of pregnancy, namely pre-eclampsia, fetal growth restriction, and stillbirth (Brosens *et al.*, 2011). Despite growing evidence that mechanical cues can direct cell differentiation and migration, there have been a limited number of studies that have investigated the role of mechanics in trophoblast biology (Wang *et al.*, 2018; Wong *et al.*, 2018).

Early in development, self-organization of the embryo, and maturation of the germ layers are known to rely on intrinsic mechanical forces and external mechanical input from the fluid surrounding the embryo (Maître *et al.*, 2016; Vining and Mooney, 2017). As development progresses, the extracellular matrix (ECM) content increases and intrinsic forces exerted by cells transition from cell–cell to cell–ECM interactions (Cosgrove *et al.*, 2016). Mechanical forces are thought to direct cell fate during organogenesis as progenitor cells differentiate into diverse specialized functions in fetal organs (Li *et al.*, 2018).

In adult cells, mechanics have been shown to influence differentiation and direct migration depending on the biophysical microenvironment the cells are cultured on (Discher *et al.*, 2005; Lv *et al.*, 2015). Naïve mesenchymal stem cells differentiate into either bone, neurons, or muscle cells when cultured *in vitro* on materials that match the stiffness of their native tissue (Engler *et al.*, 2006). A change in substrate stiffness can also induce actin cytoskeletal re-organization and contractility resulting in a change in the number of focal adhesion points and thus migration speed and direction (Plotnikov *et al.*, 2012; Bangasser *et al.*, 2017). These studies emphasize the importance of mechanics in the choice of substrate cells are cultured on, whether that is two-dimensional tissue culture polystyrene or three-dimensional (3D) hydrogels.

The stiffness of most major human tissues has been characterized and ranges from soft brain (0.2 kPa) to rigid bone ($\sim$10^6^ kPa) (Vining and Mooney, 2017). This spectrum supports and influences the cell physiology of each tissue. Research investigating effects of mechanics on embryo implantation and placentation in humans is hampered by the lack of basic knowledge of the mechanical properties at the maternal–fetal interface. Here, we used *ex vivo* atomic force microscopy (AFM) to carry out direct measurements and obtain the stiffness of human nonpregnant endometrium at the secretory phase of the menstrual cycle, first trimester decidua, and placenta.

In addition, the mechanical properties of the artificial ECM, Matrigel®, was characterized. We have previously developed a microfluidics device to study EVT migration under chemical gradients, in which the primary human EVT are embedded in 3D in Matrigel® (Abbas *et al.*, 2017). This matrix has also been used to establish organoids of both the endometrium and placenta (Boretto *et al.*, 2017; Turco *et al.*, 2017, 2018; Haider *et al.*, 2018). Matrigel® is derived from the mouse sarcoma cell line and is ~60% laminin, 30% collagen IV, and 8% entactin (Glentis *et al.*, 2014). Since mechanics is a known regulator of migration and stem cell differentiation, it is important to determine whether the stiffness of Matrigel® is physiological for trophoblast migration and stem cell studies. Direct comparisons are facilitated here by using the same techniques for Matrigel® and for human tissues.

## Materials and Methods

### Patient samples and ethics

Samples of first trimester placental and decidual tissue were taken from routine terminations of pregnancy (6–12 weeks gestation) as previously described (King *et al.*, 1989). Ethical approval for sampling placenta and decidua was obtained from the Cambridgeshire 2 Research Committee (reference no. 04/Q0108/23). Endometrial samples were taken during the secretory phase, between 7 and 10 days after the pre-ovulatory luteinizing hormone (LH) surge. Donors were recruited from the Implantation Clinic at University Hospitals Coventry and from the Warwickshire National Health Service Trust with ethical approval from National Health Service (NHS) National Research Ethics—Hammersmith and Queen Charlotte’s & Chelsea Research Ethics Committee (1997/5065). Written informed consent was obtained from all participants in accordance with the guidelines in the Declaration of Helsinki (WHO, 2000). None of the subjects were on hormonal treatments for at least 3 months prior to the procedure. Endometrial biopsies were obtained using a Wallach Endocell sampler (908014A, Wallach Surgical Devices, USA) starting from the uterine fundus and moving downward to the internal cervical os.

### Tissue preparation

**Figure 1:**
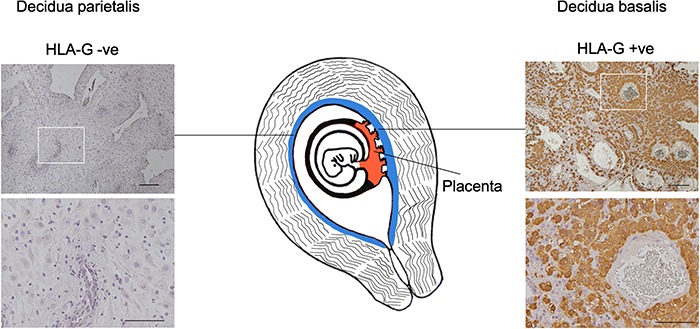
**Tissues sampled at the maternal–fetal interface during first trimester pregnancy.** Stiffness measurements were obtained using an AFM on decidua basalis, decidua parietalis, and placenta obtained at gestational ages between 8 and 10 weeks. Decidua was stained by IHC for HLA-G+ EVT to confirm whether the tissue was decidua basalis, while decidua parietalis was negative. Scale bars are 200 and 100 μm (insets).

Decidua samples were separated into suspected decidua basalis (visually by attachment of placental villi) and decidua parietalis (no placental villi) and cut into 1 × 1 × 0.5 cm pieces. This was verified post-AFM analysis using immunohistochemistry (IHC). Decidua basalis was shown to contain HLA-G+ EVT cells while decidua parietalis was negative (Fig. 1). For the placenta, a smaller cross-section of 0.5 × 0.5 × 0.3 cm was cut. Nonpregnant endometrium biopsies were received as tubes ~0.25 cm in diameter. Samples were transported at room temperature in RPMI 1640 medium (21875-034, Thermo Fisher, USA) before measurement with the AFM. All samples were embedded on a 35 mm plastic dish with the addition of 2.5% (w/v) agarose (A6103, Sigma, USA) until the edges were submerged, leaving the center exposed. RPMI 1640 medium was added until the tissues were submerged, and measurements were carried out at room temperature.

### Matrigel® preparation

The stiffness of five Matrigel® (356231, Corning, USA) batches (LOT: 6270006, 6221009, 6291008, 6214005 and 6277004) was determined. Once thawed, 20 μl drops were pipetted onto a 35 mm petri dish and placed at 37°C for 20 min to polymerize. After polymerization, the dish was filled with medium used to culture primary EVT, HAMS-F12 (P04-15549, Pan-Biotech, Germany), and all measurements were carried out at 37°C. The stiffness of Matrigel® diluted to 80% (v/v), 60% (v/v), and 40% (v/v) with EVT medium was also measured.

### Atomic force microscopy

**Figure 2:**
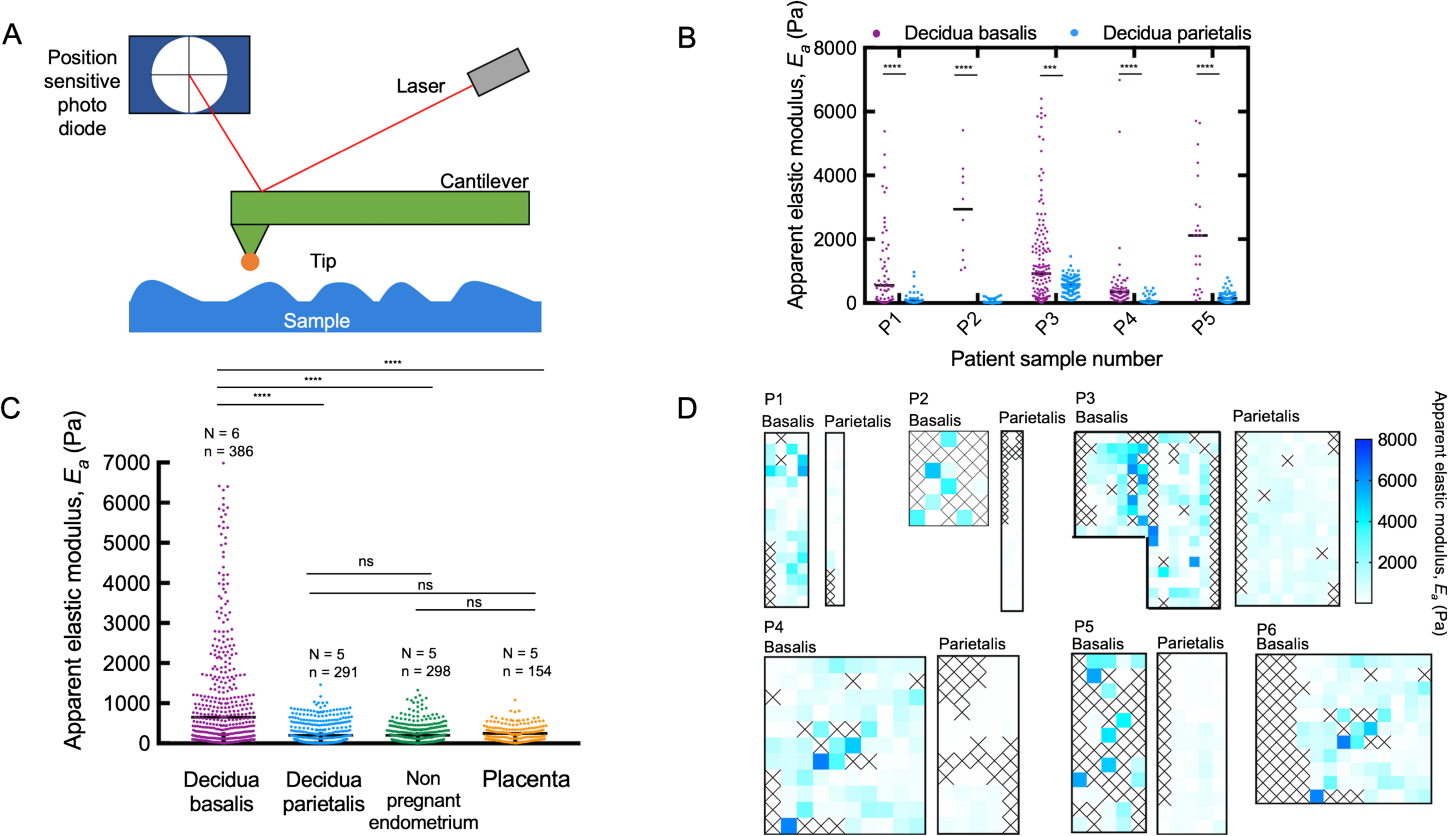
**AFM measurements showing increased stiffness of decidua basalis.** (**A**) AFM was used to determine the stiffness of samples by measuring the force between a cantilever and the sample in a feedback mechanism. The reflected laser beam strikes a position-sensitive photo diode consisting of a four segment photo detector, and this is used to determine the angular deflections of the cantilever. The Hertz model can be used to estimate the apparent elastic modulus, *E_a_*. (**B**) Decidua basalis was stiffer than decidua parietalis in five patient-matched samples (Mann–Whitney; ^****^P < 0.0001; ^***^P < 0.001; number of measurement points in each sample ranged from *n* = 10 to 148). Black bars show median for each sample. (**C**) A comparison of the apparent elastic modulus for decidua basalis, decidua parietalis, nonpregnant endometrium, and placenta villi; values measured for all samples are shown together. Black bars show median for each sample type (Kruskal–Wallis ANOVA with multiple comparison tests between groups; ^****^P < 0.001; ns means nonsignificant; *N*, number of patient samples; *n*, number of measurements). (**D**) Map of apparent elastic modulus values for decidua basalis and parietalis in paired samples**.** Each square has an area of 20 × 20μm, inside which a single measurement was taken; squares colored according to scale bar on right. Crosses represent where a clean measurement could not be taken; these are determined by evaluating the force–displacement curves manually during post-experiment analysis.

*Ex vivo* measurements and data analysis were carried out as previously described (Christ *et al.*, 2010; Koser *et al.*, 2015). Briefly, AFM was used to determine sample elasticity when exposed to indentation forces via a cantilever probe (Fig. 2A). Samples were placed on an inverted optical microscope (Axio Observer.A1, Carl Zeiss, Germany) and indentation measurements were taken with a JPK Nanowizard Cellhesion 200 AFM (JPK Instruments, Germany). Tipless silicon cantilevers (Arrow-TL1, NanoSensors, Switzerland; and SICON-TL, Applied Nanostructures, USA) with a spring constant of 0.01 to 0.1 N/m were used in these experiments. An estimate of the spring constant was calculated via the thermal noise method (Hutter and Bechhoefer, 1993) and a polystyrene bead (37.28 +/− 0.34 mm diameter; Microparticles GmbH, Germany) was glued onto each cantilever using ultraviolet curing glue (UV349, Loctite, Germany) prior to all measurements. A charged coupled device (CCD) camera (The Imaging Source GmbH, Germany) was used to image and track the position of the cantilever above the sample. This set-up was used to locate and define an area of interest on the sample on which AFM measurements were taken. A custom Python script (Python Software Foundation, USA) divided this area into 20 × 20 μm squares, inside which a single measurement was taken.

In this study, the stiffness measured is referred to as ‘apparent’ elastic modulus, since assumptions of Hertzian elastic contact do not fully apply for biological materials (Johnson, 1985). For each apparent elastic modulus measurement, the cantilever probe was lowered onto the surface of the sample at a speed of 10 μm/s. The probe continued to be lowered onto the sample until a force of 10 nN was reached. At this point the probe was retracted at a speed of 50 μm/s, the sample moved, and a measurement repeated at a different location. A threshold force of 10 nN on the cantilever-bead combination used translated to a maximum indentation depth into the sample of 1 to 5 μm.

#### Data analysis

The AFM carried out readings of cantilever z-position and cantilever angle (directly related to force applied). This produced force–displacement curves for every indentation of the sample. Using a custom MATLAB (R2008a, Mathworks, USA) script, an apparent elastic modulus value *E*_a_ was obtained from each of these curves using the Hertzian contact model (Johnson, 1985) for contact between a sphere and a half space:

$$\mathrm{F}=\frac{4}{3}{\mathrm{E}}_{\mathrm{a}}{\mathrm{R}}^{\frac{1}{2}}{\updelta}^{\frac{3}{2}}$$

where *F* is the force applied at the indentation depth $\delta$ chosen, and $R$ is the radius of the polystyrene bead. This relies on the assumptions that the stiffness of the polystyrene bead is much greater than that of the sample being measured. Polystyrene has an elastic modulus in the 10^9^ Pa range, much higher than the 10^2^ to 10^4^ Pa range covered in these experiments.

For the Hertz model to be valid, strain applied to the sample must be small and indentation depth must be much smaller than the sample thickness. Apparent elastic modulus was calculated for an indentation depth of 2$\mu$m, which resulted in a contact radius $a=\sqrt{R\delta}$, of ~6$\mu$m. Biological materials tend to begin exhibiting deviations from linearly elastic behavior at compressive strains $\epsilon =\frac{0.2a}{R}>0.02$, which places the measurements taken within an acceptable range. The condition $\frac{a}{\mathit{\mathsf{h}}}<0.1$, with ${{\mathsf{h}}}$ being the thickness of the sample analyzed, is also satisfied, which is necessary to treat the sample as a half space (Lin *et al.*, 2009).

As part of quality control, force–displacement curves were evaluated prior to analysis through the custom script. Measurements in which the force–displacement curve did not consist of a straight line (i.e. there was no linear relationship between force exerted and indentation into the tissue) were considered failed measurements. This can, for example, be caused by the probe slipping over the tissue. Due to variability in the sample preparation, some samples were observed to be more prone to such failure events.

### Immunohistochemistry

Immediately after AFM testing, decidual and endometrial samples were fixed in 10% formalin for 12 h and embedded in paraffin wax. Tissue blocks were cut into 8μm sections and dewaxed with Histoclear (HS-200, National Diagnostics, USA) followed by rehydration through gradients of ethanol to PBS. Decidual tissue was stained with an antibody against HLA-G (MEMG/1, MCA2043, BIO-RAD, USA) at 1:50 to identify EVT cells and determine whether tissue was decidua basalis or parietalis. Endometrium samples were stained with hematoxylin and eosin to confirm presence of luminal epithelium, the surface of the endometrium. Selected decidual basalis samples were also stained with rabbit polyclonal anti-fibrillin 1 (53076, Abcam, UK) and mouse anti-cytokeratin 7 (M7018, DAKO, USA) at dilutions of 1:100 and 1:200, respectively. The primary antibody was replaced with an equivalent concentration of relevant IgG for a negative control. Prior to staining, heat-induced epitope retrieval was performed in access revelation pH 6.4 buffer (MP-607-PG1, Menarini Diagnostics, UK) or access super (MP-606-PG1, Menarini Diagnostics, UK), at 125°C in an Antigen Access pressure cooker unit (MP-2008-CE, Menarini Diagnostics, UK). Sections were blocked with 2% serum (of same species in which the secondary antibody was made) in PBS. Primary antibody incubation was 30 min at room temperature, and slides were washed in PBS. Biotinylated horse anti-mouse or goat anti-rabbit secondary antibody was used, followed by Vectastain ABC-HRP reagent (PK-6100, Vectorlabs, USA) and developed with diaminobenzidine substrate (D4168, Sigma, USA). Sections were counterstained with Carazzi’s hematoxylin and mounted in glycerol/gelatin mounting medium (GG1–10, Sigma, USA).

### Single-cell RNA sequencing analysis

In order to understand the contribution of ECM components to the stiffness of decidual tissue, recently published single-cell RNA sequencing (scRNAseq) data of first trimester decidua were analyzed to identify secreted proteins. The data, outlined in Vento-Tormo *et al.*, 2018, were obtained at https://www.ebi.ac.uk under the experiment code, E-MTAB 6701 (droplet-based, 10 × genomics data) (Vento-Tormo *et al.*, 2018). The data were analyzed using the R package, Seurat (Version 2.3.4) (Satija *et al.*, 2015). Cells with <200 detected genes and >15% mitochondrial reads were removed. Normalization, clustering, and visualization of the data were performed, and clusters representing three decidual stroma (DS) populations and EVT were identified by expression of known marker genes (Vento-Tormo *et al.*, 2018). Average gene expression of each cluster was calculated using the AverageExpression function in Seurat, and scaled Log-transformed, normalized expression levels, *LogN_e_*, of specific ECM genes were plotted as heatmaps using the R package, pheatmap.

### Statistical analysis

The median of all the measurement points was calculated to provide an apparent elastic modulus value for each patient sample. The mean of all patient samples was taken to estimate apparent elastic modulus for each tissue. For Matrigel®, the median of all measured points was calculated for each batch and the mean of all batches taken to estimate its apparent elastic modulus. The normality of all data sets was tested using the D’Agostino and Pearson test. If the data were found not to follow a Gaussian distribution, nonparametric tests were used to determine statistical significance. Tissue stiffness data were analyzed using the Mann–Whitney, Wilcoxon signed-rank test, and Kruskal–Wallis ANOVA with multiple comparison tests. The *P*-values, a measure of statistical significance is given in each figure caption. Significance was set as *P* < 0.05. Statistical analysis and data plotting were carried out using Prism, Version 7 (GraphPad, USA).

## Results

In all five patient matched samples, decidua basalis was significantly stiffer than decidua parietalis (*P* < 0.05; Mann–Whitney test; *N* = 5) (Fig. 2B). The apparent elastic modulus of decidua basalis and decidua parietalis for all patients was 1250 and 171 Pa, respectively. Decidua basalis exhibited regions of increased stiffness, resulting in a wider distribution of the apparent elastic modulus measured. Of the 386 measurements, 141 were >1 kPa for basalis compared to 6 out of 291 in parietalis (Fig. 2C). Regions of increased stiffness can be seen in the stiffness maps, where each square has dimensions of 20 × 20 μm and crosses represent where a clean measurement could not be taken (Fig. 2D). Immediately after AFM testing each sample was fixed and stained by IHC with HLA-G, a unique signature of the EVT phenotype. Positive staining confirmed the tissue measured was decidua basalis whereas the negative tissue was decidua parietalis (Fig. 1).

**Figure 3:**
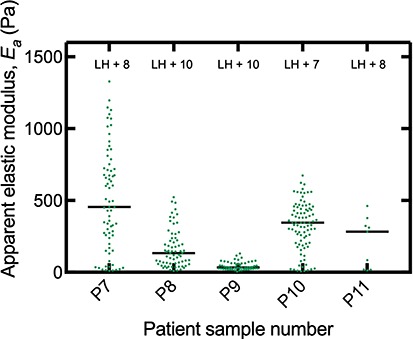
**Stiffness of individual nonpregnant endometrium patient samples**. Biopsies of secretory phase endometrium at 7–10 days after pre-ovulatory LH surge were tested using the AFM to determine apparent elastic modulus, *E_a_* of each sample (*N* = 5 and number of measurement points in each sample ranged from *n* = 9 to 89). Black bars show median for each sample. Endometrium samples were stained with hematoxylin and eosin to confirm presence of luminal epithelium.

The apparent elastic modulus of nonpregnant endometrium at the secretory phase of the menstrual cycle was found to be 250 Pa (Fig. 2C), and this was not significantly different to decidua parietalis (*P* > 0.05; Mann–Whitney; *N* = 5). There was significant variation in the range of apparent elastic modulus measured in the nonpregnant endometrium patients. This was most obvious between patient P7 with a range of 10.77 and 1328.7 Pa and patient P9 with a range of 4.8 and 131.9 Pa (Fig. 3). It is unclear whether this is due to true mechanical differences in these tissues or other factors such as location of the biopsy in the endometrium. Given these are patients attending investigations for known fertility issues, there may be pathology associated with the endometrium tested. For each nonpregnant endometrium sample, the tissue was fixed and stained with hematoxylin and eosin to confirm presence of luminal epithelium, the surface of the endometrium. There was no evidence of myometrium, the muscle layer of the uterine wall in these biopsies.

**Figure 4:**
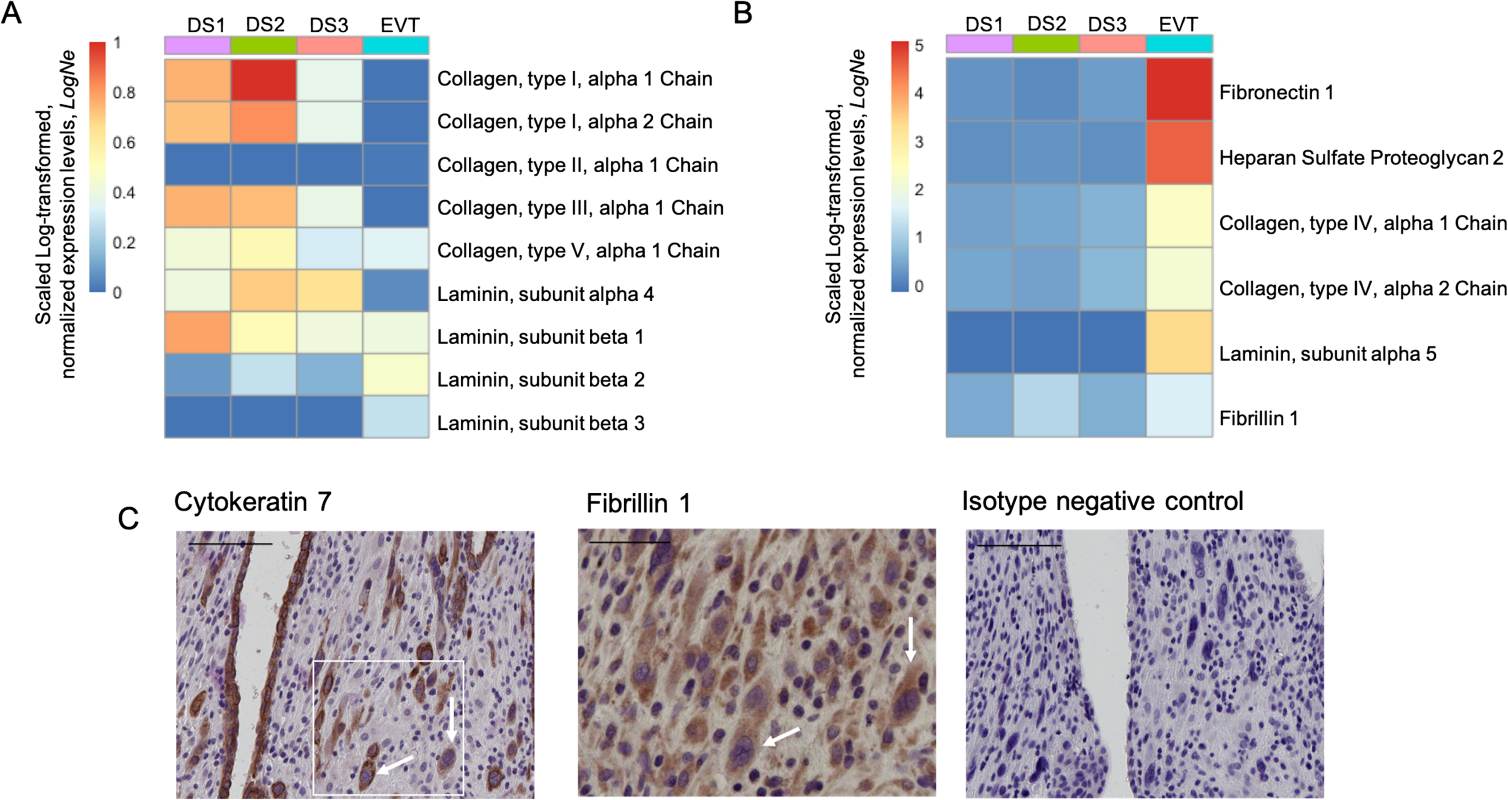
**Contribution of ECM proteins to decidual stiffness.** Analysis of single-cell RNA sequencing of first trimester decidua (data from Vento-Tormo *et al.,* 2018) show normalized expression levels of RNA transcripts encoding selected ECM components. Panel (A) shows transcripts where scaled Log-transformed, normalized expression levels, LogN_e_ in EVT $\leq$}$$ 1 compared with three stromal cell subsets: DS1, DS2, and DS3 identified by scRNAseq. Data from Vento-Tormo *et al.,* 2018. Panel (B) shows ECM transcripts where expression in EVT is more that in decidual subsets, LogN_e_$\geq$}$$1. Decidual stromal cell subsets DS1, DS2, and DS3 primarily express fibrillar collagens that provide mechanical strength. EVT cells primarily express ECM proteins that make up basement membrane. Fibrillin 1, a glycoprotein that is known to provide structural support in connective tissues, was found to be expressed by both EVT and stromal cells. (C) Protein expression of fibrillin 1 was confirmed by IHC on EVT cells. Serial sections of decidua basalis were stained for cytokeratin 7 (uterine glands and EVT) and fibrillin 1 (EVT and decidual stromal cells). Fibrillin panel shows staining of matching area to box on cytokeratin panel. White arrows indicate EVT with co-expression of both cytokeratin 7 and fibrillin 1 in serial sections (*N* = 3 biological replicates). Scale bars are 100 μm (cytokeratin panel) and 50μm (fibrillin panel).

The finding that decidua basalis is increased in stiffness compared with parietalis suggests that this is driven by the presence of invading EVT. We used a recently published scRNAseq atlas of first trimester decidua to determine the contribution of ECM proteins to decidual stiffness (Vento-Tormo *et al.*, 2018). Matrix proteins such as the fibrillar collagens type I, II, III, and V, which are known to contribute to mechanical strength (Bella and Hulmes, 2017), were produced primarily by decidua stromal cells (Fig. 4A). In contrast, EVT cells predominantly make ECM proteins that contribute to the formation of basement membrane (Fig. 4B) such as collagen, type IV, fibronectin, laminin, and heparan sulfate (Lowe *et al.*, 2015). This analysis suggests production of fibrillin 1 by EVT isolated in the decidua. Fibrillin 1 is a major component of microfibrils and provides structural support in a large number of connective tissues. Using IHC on serial sections of decidua basalis, we confirmed localization of fibrillin 1 to cytokeratin 7+ EVT and to some stromal cells (Fig. 4C).

The placenta is the extraembryonic organ that supports the growth and development of the fetus. In the first trimester, it is exposed to secretions by decidual glands before mediating transport of nutrients from maternal blood when this supply is established at ~11 weeks’ gestation. The apparent elastic modulus of placental villi samples from gestational ages 8–10 weeks was found to be 232 Pa (Fig. 2C).

**Figure 5:**
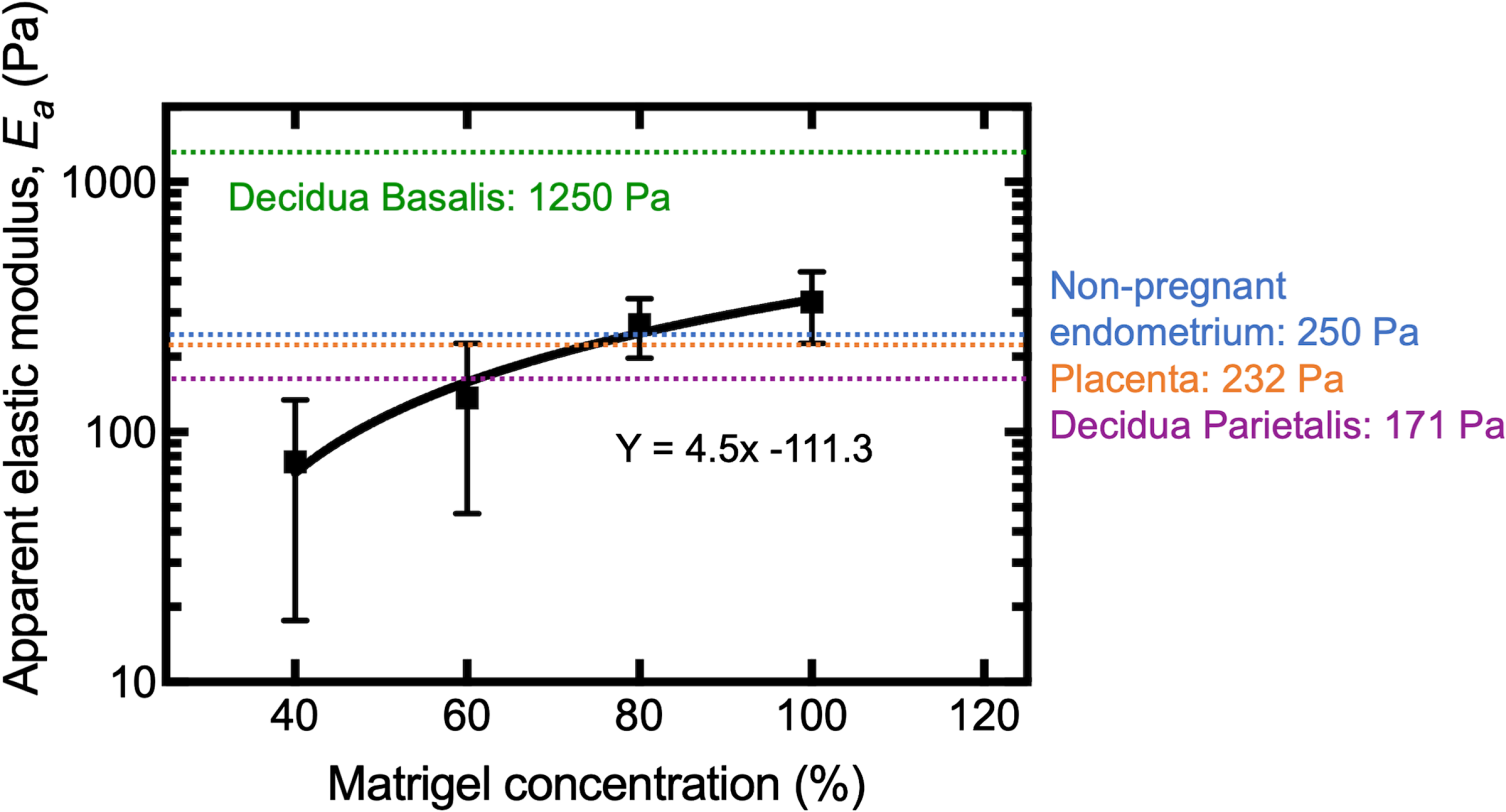
**An order of magnitude difference in apparent elastic modulus between Matrigel® and decidua basalis.** AFM was used to determine stiffness of five batches of 100% Matrigel® (undiluted) and at dilutions to 80%, 60%, and 40% with EVT medium. Matrigel® is an artificial ECM basement membrane and the most common substrate used in 3D migration assays with EVT and culture of organoids. Nonlinear regression analysis was used to fit the data and determine the equation of the line. Matrigel® stiffness was similar to that for endometrium and first trimester placenta but much lower than that for decidua basalis.

Matrigel® is used for 3D organoid cultures of endometrium and placenta, as well as trophoblast migration assays (Abbas *et al.*, 2017; Boretto *et al.*, 2017; Turco *et al.*, 2017, 2018; Haider *et al.*, 2018). The stiffness of five batches of Matrigel® was therefore determined using the AFM (Fig. 5). For pure or 100% Matrigel® the apparent elastic modulus was found to be 331 Pa, significantly lower than for decidua basalis (*P* < 0.05; Mann–Whitney test; *N* = 5). Stiffness was determined for Matrigel® diluted to 80%, 60%, and 40% with medium used to culture primary EVT (HAMS-F12). A linear regression analysis was performed with R^2^ = 0.97 and equation of the line Y = 4.5x − 111.3, which allows the effects of Matrigel® dilution to be estimated. Different batches were tested because Matrigel® is not a well-defined matrix, which can be a source of variability (Gjorevski *et al.*, 2016). The range of apparent elastic modulus found for pure 100% Matrigel® was between 243 and 512 Pa, showing significant batch variation.

## Discussion

Proper invasion of the decidua by EVT during early pregnancy is critical for successful placentation. Although mechanical cues are known to be important in regulating cell differentiation and cell migration, their role in differentiation of trophoblast stem cells and invasion of EVT into the decidua has been neglected. We have used AFM indentation to carry out direct measurements and obtain the first measurements of stiffness of human nonpregnant endometrium, first trimester decidua, and placenta.

We found that the stiffness of nonpregnant endometrium, decidua parietalis, and placenta are at a magnitude of 10^2^ Pa and similar to that of neural tissue (Vining and Mooney, 2017). By contrast, decidua basalis is an order of magnitude stiffer at 10^3^ Pa and similar to that of breast tissue (Butcher *et al.*, 2009). The finding that pre-decidualized endometrium and fully decidualized decidua parietalis are similar suggests that changes in ECM that are known to accompany decidualization do not drive the change in tissue stiffness (Gellersen and Brosens, 2014). The increase in stiffness in decidua basalis is therefore likely to be due to changes induced as a result of EVT invasion. EVT cells invade the decidua in a controlled and directed manner and remodel the spiral arteries, converting them into highly dilated vessels capable of providing sufficient nutrients and oxygen to the fetus (Jauniaux *et al.*, 2000). As EVT invade, they degrade stromal ECM proteins while at the same time producing their own distinct ECM networks (Burrows *et al.*, 1996; Zhu *et al.*, 2012).

The stiffness of tissues is correlated with the type and quantity of ECM proteins (Handorf *et al.*, 2015). Therefore, it is important to ask, what ECM proteins do EVT cells deposit in the decidua? Fibronectin is a major component of EVT matrix production, but this is typically found in basement membrane and is not associated with tissue stiffening (Burrows *et al.*, 1993). We have previously shown EVT cells produce collagen IV both *in vivo* and *in vitro* (Oefner *et al.*, 2015). Collagen IV is abundant in the lamina densa of all basement membranes but it is typically non-fiber-forming (Timpl and Dziadek, 1986; Schwarzbauer, 1999). In the placental villous mesenchyme, it occurs in a complex 3D network encapsulating stromal cells. At the implantation site, it is densely deposited in a non-fibrous form by EVT and is unlikely to contribute to tissue stiffness. Recent scRNAseq of first trimester decidua has suggested other ECM proteins including fibrillin 1 are synthesized by EVT, and we confirmed this by immunostaining (Fig. 4). Fibrillin 1 is a glycoprotein that assembles to form 10–12 nm microfibrils in ECM (Handford, 2000). Its function is to provide both structural support and homeostasis through specific interactions with growth factors and it is found typically in connective tissues (Zhang *et al.*, 1995; Handford, 2000). It contributes to increased tissue stiffness by acting as a fiber-reinforced composite, with the evidence being primarily in elastin rich tissues (Sherratt *et al.*, 2003). Fibrillin 1 expression has previously been reported to increase between the luteal phase endometrium and first trimester decidua (Fleming and Bell, 1997).

The decidua is unusual in that an increase in stiffness is not correlated with an increase in structural ECM, such as collagen I (Oefner *et al.*, 2015). Although the ECM proteins produced by EVT, fibronectin, collagen IV, heparan sulfate, and fibrillin 1 are not typically associated with stiffening, the combination of these proteins may have an impact. The endometrium undergoes extensive remodeling during decidualization and invasion by EVT. We found areas of increased stiffness, demonstrated by the stiffness maps, which supports the idea of local differences in decidua basalis. A likely explanation is the presence of the highly coiled spiral arteries that run perpendicularly toward the uterine surface, to supply the placenta. In the nonpregnant state, these arteries contain large quantities of smooth muscle in their walls and are surrounded by densely packed stromal cells. Together these form structures that have been referred to as Streeter’s columns, which are 2–3 mm apart (Oefner *et al.*, 2015). Given that the contact diameter of the cantilever tip was calculated to be 12 μm, the measured local stiffness results will vary with proximity to the columns. During early pregnancy, EVT cluster around the arteries and induce their remodeling, whereby dedifferentiation of the smooth muscle cells is accompanied by the deposition of an inert fibrinoid material. Measurements taken on or close to a column are therefore likely to yield stiffer results compared to counterparts taken over the glandular interstices. The necrotic residues of decidual and trophoblast cells that are transformed into fibrinoid, known as the ‘Nitabuch layer’, may also contribute to the wide range of stiffness values in decidua basalis (Benirschke *et al.*, 2012).

Local differences in stiffness may influence EVT migration, and the most dramatic change in stiffness EVT cells will encounter is when they reach the myometrium, the muscle layer underneath the decidua. The stiffness of smooth muscle tissue is ~5 kPa, five times that of decidua basalis (Butcher *et al.*, 2009). In normal pregnancies EVT cells stop migrating once they reach the inner third of the myometrium where they terminally differentiate into multinucleated giant cells.

One limitation of AFM in dense tissue is the inability to localize stiffness measurements to location in the tissue. Clearing techniques may be used to improve microscopy; however, this would require fixation and time resulting in likely changes to tissue mechanics. Differences in shape of tissue blocks sampled by AFM can also affect stiffness measurements. Endometrial biopsies are tubular while decidual samples tended to be cuboidal. Curved surfaces can be taken into account in the analysis but this was unnecessary here, as the large radius of curvature of the samples compared to that of the cantilever probe bead made this contribution negligible.

Finally, Matrigel® is the most common substrate used to study EVT migration *in vitro* in 3D and more recently it has been used to establish organoids of the placenta and endometrium (Boretto *et al.*, 2017; Turco *et al.*, 2017, 2018; Haider *et al.*, 2018). We found that Matrigel® was approximately an order of magnitude softer than decidua basalis and is not physiological for investigating EVT migration into decidual basalis. In contrast, Matrigel® is within the range of measurements for both nonpregnant secretory phase endometrium and placenta, which makes it appropriate to study early blastocyst implantation and may explain its successful use as a matrix for growth in organoid cultures of both these tissues. Diluting the Matrigel® concentration can be used to tune stiffness to match a desired tissue. However, a major disadvantage is the batch-to-batch variation in protein content. We found the stiffness measured for five batches had a wide range from 243 to 512 Pa, which is in agreement with measurements of Matrigel® previously made with an AFM (Soofi *et al.*, 2009). Long term, a move is needed to more synthetic substrates with defined ECM content and stiffness to ensure consistency between experiments. The composition of these substrates could be adjusted to reflect more closely the stiffness of the decidua.

### Summary

Mechanics is now widely recognized as an important regulator of cell behavior. However, studies on the influence of mechanics on blastocyst implantation and development of the placenta have been lacking. Here, we found the stiffness of nonpregnant endometrium, decidua parietalis, and placenta to be ~10^2^ Pa compared to 10^3^ Pa for decidua basalis. The data generated here can be used as a basis for future research on mechanics at the maternal–fetal interface and to optimize 3D culture and migration assays currently being developed to more closely mimic the real tissue environment.
